# Prognostic value of the immunohistochemical detection of epithelial-mesenchymal transition biomarkers in oral epithelial dysplasia: A systematic review

**DOI:** 10.4317/medoral.23305

**Published:** 2020-01-22

**Authors:** Everton Freitas de Morais, Juliana Campos Pinheiro, Jadson Alexandre Silva Lira, Rodrigo Porpino Mafra, Carlos Augusto Galvão Barboza, Lélia Batista de Souza, Roseana de Almeida Freitas

**Affiliations:** 1 DDS, MSc, PhD Student, Department of Oral Pathology, Federal University of Rio Grande do Norte, Natal, Rio Grande do Norte, Brazil; 2 DDS, MSc, PhD Student, Department of Morphology, Federal University of Rio Grande do Norte, Natal, Rio Grande do Norte, Brazil; 3 DDS, MSc Student, Department of Morphology, Federal University of Rio Grande do Norte, Natal, Rio Grande do Norte, Brazil; 4 DDS, MSc, PhD, Professor, Morphology, Federal University of Rio Grande do Norte, Natal, Rio Grande do Norte, Brazil; 5 DDS, MSc, PhD, Professor, Department of Oral Pathology, Federal University of Rio Grande do Norte, Natal, Rio Grande do Norte, Brazil

## Abstract

**Background:**

Oral potentially malignant disorders (OPMDs) comprise a range of clinical-pathological alterations that are frequently characterized as architectural and cytological derangements upon histological analysis. Epithelial-mesenchymal transition (EMT) has been proposed as a critical mechanism for the acquisition of the malignant phenotype in neoplastic epithelial processes. This study aims to systematically review the current findings on the immunohistochemical expression of epithelial-mesenchymal transition markers in oral potentially malignant disorders and to evaluate their possible application as biomarkers associated with the progression of oral epithelial dysplasias.

**Material and Methods:**

A systematic search was performed in the following databases: PubMed, EMBASE, Chinese BioMedical Literature Database, and Cochrane Library. Articles that evaluated the relationship between the expression of EMT markers and the degree of oral epithelial dysplasia were selected for the systematic review. The quality of each eligible study was evaluated by independent reviewers that used operationalized prognostic biomarker reporting guidelines (REMARK).

**Results:**

Seventeen articles met all inclusion criteria and were selected. The EMT markers analyzed exhibited an important association with the prognosis of the cases evaluated. The results showed a progressive increase in the expression of nuclear transcription factors and markers of mesenchymal differentiation, as well as negative regulation of epithelial and cell adhesion markers, according to the stage of oral epithelial dysplasia.

**Conclusions:**

The dysregulation of expression of important EMT components in oral dysplastic epithelium is a potential prognostic marker in OPMDs.

** Key words:**Oral potentially malignant disorder, oral epithelial dysplasia, epithelial-mesenchymal transition, biomarker, prognosis.

## Introduction

Oral potentially malignant disorders (OPMDs) comprise a range of clinical-pathological alterations that are frequently characterized as architectural and cytological derangements upon histological analysis (1-3). OPMDs exhibit an increased risk of malignant transformation. A recent meta-analysis estimated an overall risk of malignant transformation of 10.5% for oral epithelial dysplasia (4).

The potential of malignant transformation is believed to be related to the degree of epithelial dysplasia observed (2-4). Several studies have been conducted to identify possible markers that can trigger the development of OPMDs as well as predict their progression (2,5,6). The epithelial-mesenchymal transition (EMT) is a mechanism frequently dysregulated in cancer (5-10,12). However, studies evaluating the role of EMT in the development/progression of dysplastic processes in oral epithelium are scarce.

The EMT, which is an essential event during embryogenesis, is a biological process in which epithelial cells lose their characteristics and shift to a mesenchymal cell-like phenotype. This process has been proposed as a critical mechanism for the acquisition of the malignant phenotype in neoplastic epithelial processes (5,8,9,12). EMT is mediated by nuclear transcription factors and can orchestrate intracellular alterations such as the negative regulation of epithelial markers, as well as the positive regulation of some mesenchymal markers (7,8,10).

A variety of biomarkers are known to be associated with EMT. These biomarkers can be divided into five groups according to their characteristics/functions: 1. cell surface markers; 2. cytoskeletal markers; 3. extracellular proteins; 4. transcription factors; 5. epigenetic markers and microRNAs.8 Recent studies indicate that important EMT markers may be involved in the malignant transformation of cases diagnosed as OPMD (5,11,12).

In this study, a systematic review was conducted to clarify the prognostic value of EMT biomarkers in OPMDs. The review includes publications that evaluated the immunodetection of EMT markers and their possible use as prognostic factors/predictors in oral epithelial dysplasia.

## Material and Methods

We performed a systematic review to conduct this investigation. The dependent variables were biomarkers of EMT available by immunohistochemistry and the independent variables were clinicopathological parameters and oral epithelial dysplasia grading. The Reporting Items for Systematic Reviews and Meta-Analyses (PRISMA) guidelines were followed (13).

- Search strategy

The research question was “Are epithelial-mesenchymal transition biomarkers analyzed by immunohistochemistry potential predictors/prognostic factors for oral epithelial dysplasia?” and a keyword search was performed.

To identify all primary research articles that evaluated EMT biomarkers in oral epithelial dysplasia, we searched the MEDLINE/PubMed (1966 to January 2019), EMBASE (1980 to January 2019), Cochrane Collaboration Library (2009 to January 2019), and Chinese BioMedical Literature Databases (1978 to January 2019). The search strategy was based on combinations of the following keywords: (“Oral epithelial dysplasia” [MeSH] AND “Immunohistochemistry” [MeSH] AND “biomarkers of epithelial-mesenchymal transition” [MeSH] AND “clinicopathological parameters” [MeSH] AND “outcome” [MeSH]) AND (risk ratio [Title/Abstract] OR relative risk [Title/Abstract] OR odds ratio [Title/Abstract] OR risk [Title/ Abstract]) AND (“humans”[MeSH Terms]). A manual search of articles was also performed using the references within studies with inclusion potential in the systematic review.

- Selection criteria

Articles were included based on a previously published protocol (14,15). Studies that assessed the relationship between the immunohistochemical expression of EMT markers and histopathological grading of cases diagnosed with oral epithelial dysplasia were selected. The search was carried out without time and language restrictions. The PICOS (population, intervention, comparison, outcome, study design) format was used to construct the research question using the following inclusion criteria: (I) Population: patients diagnosed with oral epithelial dysplasia; (II) Intervention: immunohistochemical analysis of EMT markers; (III) Outcome: risk of progression from oral epithelial dysplasia; (IV) Study Design: observational studies in humans.

We limited selection to human studies on oral epithelial dysplasia defined based on standardized histological assessment as outlined by the WHO (16). We included all studies that reported data for progressing and non-progressing oral epithelial dysplasias. Both prospective and retrospective studies were included. Progressing lesions were defined as those dysplasias that developed cancer at the same site as the initial biopsy when followed over time. Biomarkers of EMT were defined according to the study by Zeisberg & Neilson (8).

In the screening of titles or abstracts, citations were retained if they were original studies, except for reviews or meeting reports, that explored associations between the immunoexpression of the EMT markers, clinicopathological characteristics, and outcome/progression of the oral epithelial dysplasia cases analyzed. The articles were selected independently by two reviewers (EFM and JCP). Any disagreement was resolved by consensus.

In the final full-text screening, studies were included if they met the following criteria: (I) designed as a prospective or retrospective cohort study; (II) oral epithelial dysplasia was diagnosed and histopathological grading was performed by pathological examination; (III) data about the association between the EMT biomarker evaluated and histopathological grading were reported. When studies were based on overlapping data, the more comprehensive set was selected.

- Data extraction and analysis

Quality assessment was performed in duplicate for each eligible study by three independent reviewers using the Newcastle operationalized prognostic biomarker reporting guidelines (REMARK) (15). Any disagreement was resolved by consensus. Studies receiving a score of less than 6 were not included in the systematic review. Three reviewers (EFM, JASL, RPM) independently extracted the data from the selected articles.

- Risk of bias in individual studiesMethodologically, the authors appraised all of the included studies according to a checklist based in Meta -Analysis of Statistics Assessment and Review Instrument (MAStARI) (17). Two reviewers (EFM and RPM) answered 9 questions for descriptive studies as Y for “yes”, N for “no”, U for “unclear” and NA for “not applicable”. After that, the risk of bias was categorized as high when the study reached up to 49% of a “yes” score, moderate when the study reached 50% to 69% of a “yes” score, and low when the study reached more than 70% of a “yes” score. Disagreements were solved by discussion between the four authors (EFM, RPM, JCP, JASL).

## Results

- Study selection and characteristics

**Figure 1:**
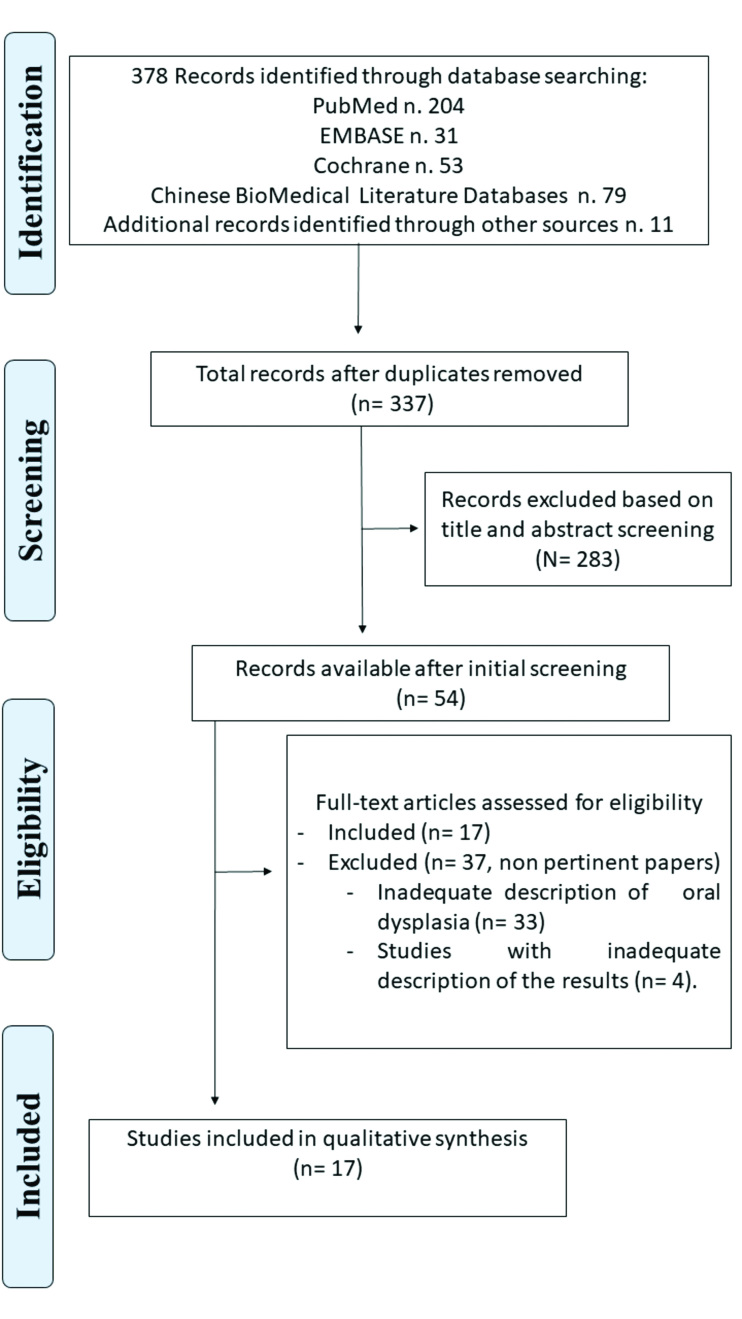
PRISMA flow diagram of screened studies.

The database searches and manual search developed in this systematic review retrieved 378 studies. After reading the titles and abstracts, 54 studies were considered potentially eligible and their full text was read by three reviewers. Seventeen articles m*et al*l inclusion criteria and were selected for this systematic review (5,18-33). Fig. 1 illustrates the flow diagram of the screening and selection process of the articles.

The selected articles were observational studies published between 2007 and 2018, all of them in English. Regarding methodological characteristics, all articles included in this systematic review involved 1,178 patients, with a mean of 69.2 participants per study. Our analysis revealed that 743 (63%) of the cases evaluated were lesions diagnosed in different stages of epithelial dysplasia. Among these cases, 48.7% were graded as mild epithelial dysplasia and 51.3% as moderate or severe epithelial dysplasia. The main characteristics and findings of the studies are shown in Table 1.

Most of the epithelial dysplasia cases were clinically diagnosed as leukoplakia and/or erythroplakia. Anura *et al*. (25) and Sharada *et al*. (33) also evaluated tissue samples of oral submucosal fibrosis. Twelve studies included samples of oral squamous cell carcinoma (OSCC) for biomarker analysis, totaling 426 cases analyzed (5,18,21,23,24,26-28,31-33).

**Table 1:**
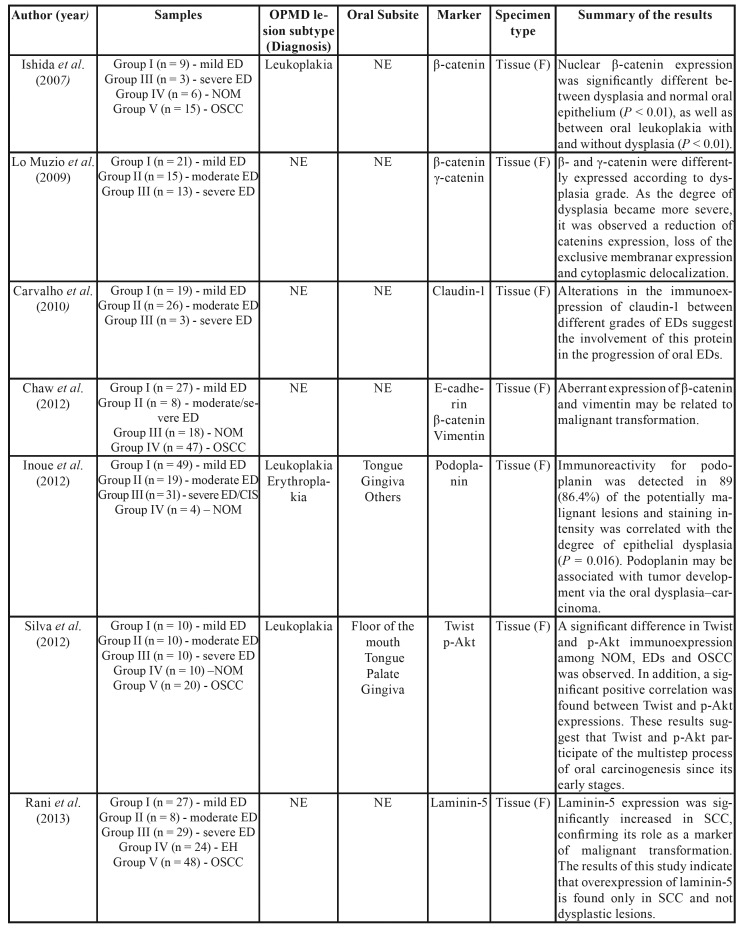
Summary of the descriptive characteristics and results of the included studies (n=17).

**Table 1 cont.**
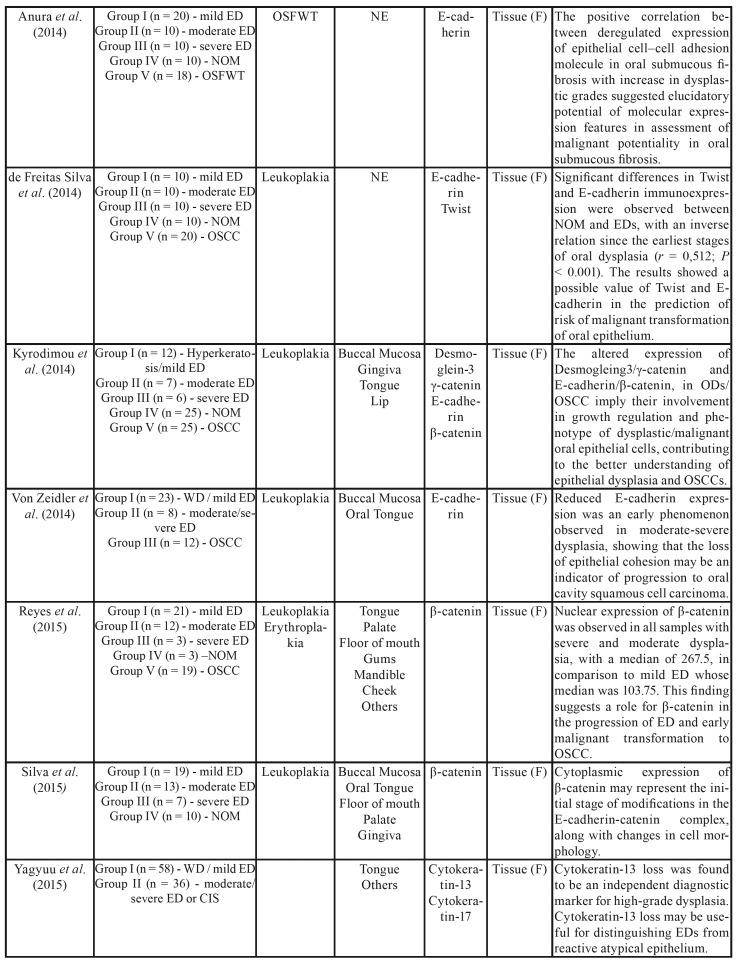
Summary of the descriptive characteristics and results of the included studies (n=17).

**Table 1 cont.**
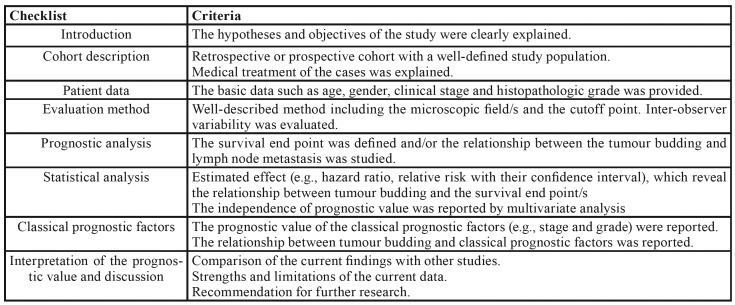
Summary of the descriptive characteristics and results of the included studies (n=17).

The following EMT markers were analyzed according to the criteria proposed by Zeisberg & Neilson8: β-catenin (18,19,26,28,29), γ-catenin (19,26), claudin-1 (20), E-cadherin (5,21,26,27,32,33), Twist (5,23), p-Akt (23), podoplanin (22), laminin-5 (24), desmoglein-3 (26), cytokeratin 13 and cytokeratin 17 (30), and N-cadherin (31). The methods used for immunohistochemical analysis in the different studies selected were identified in this systematic review (Table 2).

**Table 2:**
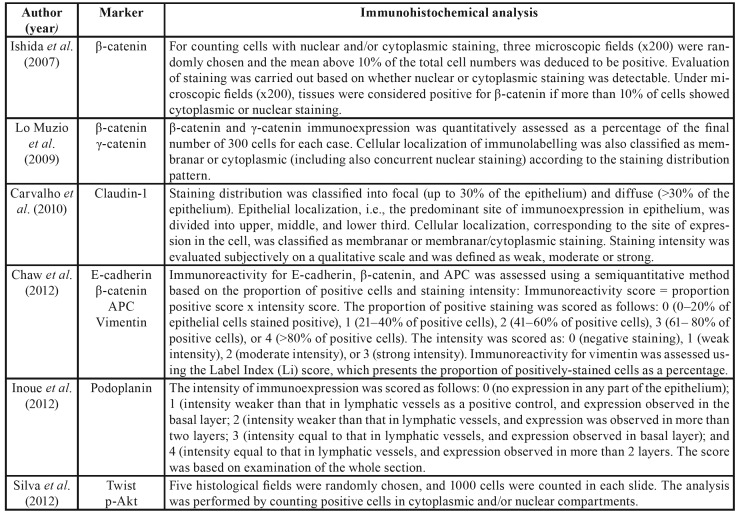
Immunohistochemical analysis used in the selected studies.

**Table 2 cont.**
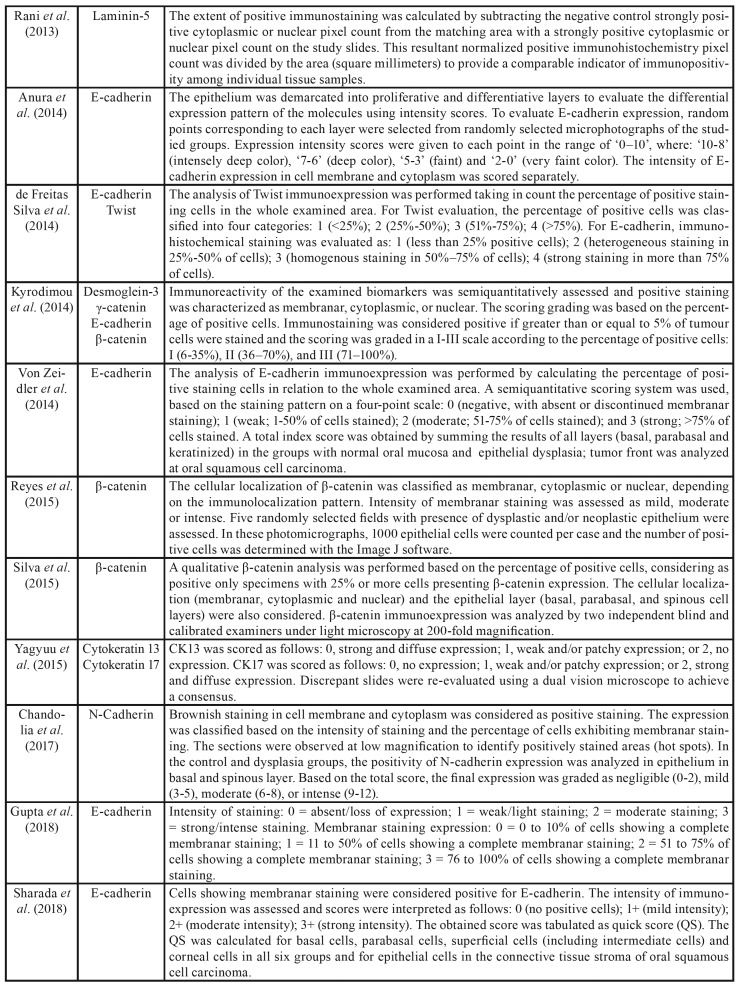
Immunohistochemical analysis used in the selected studies.

- Quality assessment and Risk of bias

We used the criteria established by the REMARK guidelines to evaluate the studies included in the systematic review (Table 3). The selected studies provided details about the objective/hypothesis of the study, characteristics of the patients included in the sample, analysis method used, and relationship of the EMT markers with the degree of epithelial dysplasia in the cases analyzed. In addition, the studies discussed implications for future studies and clinical value.

Based on the MAStARI assessment, 2 articles (18,32) were classified as carrying a high risk of bias, mainly because the answers for questions 3 and 4 (related to co -founding factors, description of the groups and follow-up, respectively) were “No”. Fifteen studies were classified as with low risk for bias (5,19-22,25,26-31,33) (Table 4).

**Table 3:**
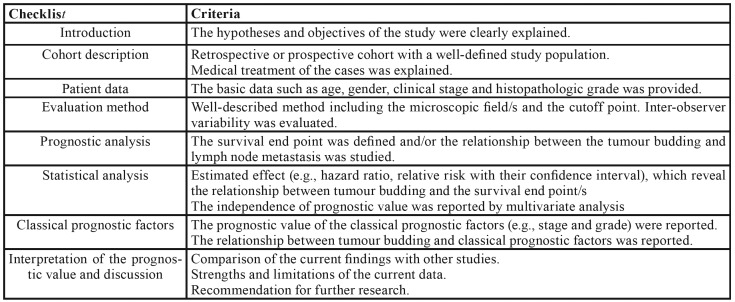
Evaluation criteria used to assess the quality of studies evaluated (adapted from REMARK guidelines).

**Table 4:**
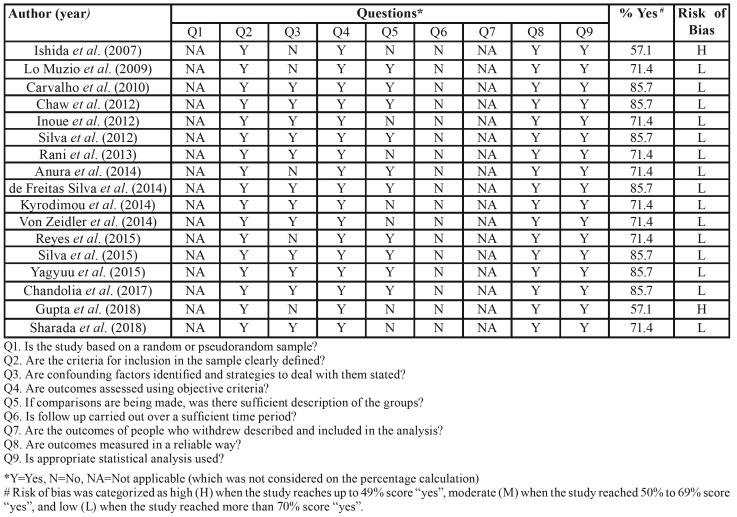
Analysis of the risk of bias of the articles included in the review was performed with the MAStARI (Meta-Analysis of Statistics Assessment and Review Instrument) critical appraisal tool.

- Cell-surface and cytoskeletal markers of the epithelial-mesenchymal transition

Among the 17 selected studies, 15 evaluated cell surface and/or cytoskeletal markers (5,18-22,25-33). These studies demonstrated significant dysregulation of the expression of the proteins analyzed according to the degree of dysplasia.

In our systematic review, β-catenin was a biomarker strongly associated with the progression of oral epithelial dysplasia. Ishida *et al*. (18) detected strong immunoexpression of β-catenin in the cell nucleus of cases diagnosed as oral epithelial dysplasia, with a significant difference between dysplasia samples and normal oral epithelium (*P* < 0.01) and between oral leukoplakia with dysplasia and the group without dysplastic alterations (*P* < 0.01). Lo Muzio *et al*. (19) observed the loss of membrane expression of β- and γ-catenin with increasing degree of oral epithelial dysplasia and a shift of expression to the cytoplasm. The same finding was reported in the other studies included (21,29).

The loss of membrane expression of cell adhesion markers and the translocation of immunopositivity to the cytoplasm was a common finding and was strongly associated with an increase in the degree of oral epithelial dysplasia. Carvalho *et al*. (20) demonstrated that all cases of mild epithelial dysplasia and 73.1% of cases of moderate epithelial dysplasia exhibited only membrane immunopositivity, while membrane/cytoplasmic staining was observed in cases of severe epithelial dysplasia. These findings show the dysregulation of protein expression with increasing degree of dysplasia.

Chaw *et al*. (21) observed a significant correlation between the loss of E-cadherin expression and increased vimentin expression in the cytoplasm in moderate/severe epithelial dysplasias. The expression of vimentin was also positively correlated with the cytoplasmic and nuclear expression of β-catenin (r = 0.467, *P* < 0.05). De Freitas Silva *et al*. (5) demonstrated a gradual loss of E-cadherin expression with increasing degree of dysplasia of the oral epithelium.

- Extracellular proteins and transcription factors

Although recognized as important markers associated with the EMT process, studies analyzing the role of transcription factors and extracellular proteins in oral epithelial dysplasia by immunohistochemistry are scarce. Silva *et al*. (23) reported a significant variation in the Twist transcription factor among five groups analyzed. The authors observed higher immunopositivity in cases of severe epithelial dysplasia compared to moderate dysplasia (*P* = 0.047) and a progressive increase of expression in cases of OSCC compared to severe epithelial dysplasia (*P* = 0.007).

In the study of De Freitas Silva *et al*. (5) immunostaining for the Twist protein was mainly observed in the parabasal and basal layers of the normal oral epithelium and in the groups with mild and moderate dysplasia, exhibiting a predominant cytoplasmic localization. However, in cases of severe epithelial dysplasia, immunoexpression was also detected in the superficial layers of the epithelium, indicating dysregulation of protein expression in advanced stages of epithelial dysplasia. The authors also showed a statistically significant correlation in the immunoexpression of Twist and E-cadherin, with the observation of an inverse relationship between the immunoexpression of these proteins (r = 0.512; *P* < 0.001).

Rani *et al*. (24) observed low expression of laminin-5 in cases of epithelial dysplasia, with predominantly weak and cytoplasmic immunostaining. In that study, the expression of laminin-5 did not differ significantly according to the degree of dysplasia. A significant increase in laminin-5 immunopositivity was only found in cases of OSCC compared to the epithelial dysplasia groups, suggesting a role of this protein in the invasion of the already established neoplastic process.

## Discussion

The present study analyzed the role of EMT in cases diagnosed with oral epithelial dysplasia. The malignant transformation of OPMDs is an important process in oral carcinogenesis, which is still poorly understood. Thus, the identification of markers that are associated with the development and progression of oral epithelial dysplasia is of paramount importance for establishing a possible relationship with the prognosis of these lesions. The histopathological classification of oral epithelial dysplasia can assist in the monitoring and definition of the most appropriate treatment; however, other factors may be determinant in the process of malignant transformation (34,35).

Several biomarkers are being analyzed by immunohistochemistry in order to evaluate their participation in oral epithelial dysplasia (3,6). In clinicopathological practice, immunohistochemistry is relatively easy to apply and more accessible than other techniques.

The EMT has been indicated as an important process in carcinogenesis that is directly associated with the aggressiveness of OSCC. During this process, epithelial cells lose their capacity of cell-cell adhesion. In addition, reorganization of the cytoskeleton and significant changes in signaling occur that define the shape and structure of the neoplastic cell. EMT is believed to be the result of reprogramming of gene expression mediated by transcription factors such as Twist, Snail, and Slug (10,36-38). This transition increases cell motility and enables the development of an invasive phenotype (37). Despite advances in the understanding of the function of EMT in already established OSCC, it is necessary to identify the role of EMT markers in the development and progression of oral epithelial dysplasia.

The loss of cell adhesion is likely to play a key role in EMT. This phenomenon can be observed in our systematic review by the loss of membrane expression of cell adhesion markers such as claudin-1 and, particularly, E-cadherin in cases diagnosed as epithelial dysplasia (5,10,21,26,27,32,33,36,38). Furthermore, E-cadherin was immunoexpressed in the cytoplasm of epithelial dysplasia cases. In this respect, studies have shown that the cytoplasmic expression of this protein is frequently associated with tumors in advanced stages (39,40). It is possible that the loss of membrane expression and the onset of E-cadherin translocation to the cytoplasm, and consequently the loss of basic functions associated with cell adhesion, occur during the early stages of oral carcinogenesis and are already present in cases of oral epithelial dysplasia, progressing with increasing severity of the latter.

Twist is an important nuclear transcription factor for EMT. De Freitas Silva *et al*. (5) indicated a repressive effect of Twist on the expression of E-cadherin in epithelial dysplasia and oral cancer. Furthermore, previous studies have elucidated the role of Twist in the already established neoplastic process, which negatively regulates the expression of E-cadherin and the overexpression of mesenchymal markers (5,10,37). Other transcription factors with a potential role in the development and progression of oral epithelial dysplasia need to be better investigated. Zheng *et al*. (41) reported the overexpression of the transcription factors Snail and Slug to be associated with a poor prognosis of the neoplastic process. The Twist, Snail and Slug proteins seem to a play a similar role in the repression of E-cadherin and, apparently, the expression of these proteins is also related to the regulation of β-catenin (42).

The binding of E-cadherin to β-catenin in the cytoplasm/membrane represses tumor progression, maintaining cell-cell adhesion and inhibiting EMT, cell motility and tumor metastasis (42). The negative regulation or loss of E-cadherin and β-catenin expression, as well as the immunoexpression of β-catenin in the nucleus, is frequently observed in several type of cancer, including head and neck cancer. In our systematic review, the nuclear expression of β-catenin was a frequent finding in the samples of cases diagnosed as advanced oral epithelial dysplasia and might be an important marker associated with the progression of this condition (18,21,28).

Prgomet, Andersson, & Lindberg44 evaluated the expression of WNT5A, β-catenin and E-cadherin by immunohistochemistry in 21 tissue samples. Each sample contained areas of mucosa with normal appearance, oral epithelial dysplasia, and OSCC. In that study, membrane expression of β-catenin was lower in OSCCs than in dysplasia or normal-appearing mucosa regions, while cytoplasmic expression of β-catenin increased with the severity of dysplasia and was detected in half of the OSCCs. Similar findings of reduced membrane expression of β-catenin and progression of oral carcinogenesis were reported in the studies included in our systematic review. The cytoplasmic accumulation and subsequent nuclear translocation of β-catenin might be the result of activation of the canonical Wnt signaling pathway or impairment of this pathway due to mutations in some of its components. The cytoplasmic/nuclear overexpression of β-catenin is known to be associated with malignant transformation in different types of cancer (28,45).

Recent *in vitro* studies confirm the role of EMT markers in the progression of oral epithelial dysplasia. Dmello *et al*. (46) evaluated the expression of vimentin in epithelial cells derived from OPMDs. In that study, the exogenous expression of vimentin contributed to the occurrence of EMT and subsequent malignant transformation. Epigenetic markers may regulate EMT. Members of the miR-200 family have been shown to be negatively regulated in human cancer cell lines and play a critical role in the suppression of EMT, tumor cell adhesion, migration, invasion, and metastasis (47). Arunkumar *et al*. (48) demonstrated expressive negative regulation of miR-200 in poorly differentiated OSCC by RT-qPCR analysis (*P* = 0.0067). However, there are no studies investigating epigenetic markers associated with EMT in oral epithelial dysplasia.

## Conclusions

In conclusion, the dysregulation of expression of EMT-associated proteins in dysplastic oral epithelium is a potential prognostic marker. The results showed a progressive increase in the expression of nuclear transcription factors and markers of mesenchymal differentiation, as well as negative regulation of epithelial and cell adhesion markers, according to the severity of oral epithelial dysplasia. It should be noted that the studies included in this systematic review examined OPMDs that were not continuously followed up from the time of diagnosis to malignant transformation. Longitudinal studies are necessary to identify the possible association of EMT with the risk of malignant transformation of OPMDs.
